# Fundamentals of the logarithmic measure for revealing multimodal diffusion

**DOI:** 10.1016/j.bpj.2021.01.001

**Published:** 2021-01-14

**Authors:** Benjamin A. Dalton, Ivo F. Sbalzarini, Itsuo Hanasaki

**Affiliations:** 1 Max Planck Institute for the Physics of Complex Systems, Dresden, Germany; 2 Technische Universität Dresden, Faculty of Computer Science, Dresden, Germany; 3 Institute of Engineering, Tokyo University of Agriculture and Technology, Koganei, Tokyo, Japan; 4 Max Planck Institute of Molecular Cell Biology and Genetics, Dresden, Germany; 5 Center for Systems Biology Dresden, Dresden, Germany; 6 Cluster of Excellence Physics of Life, TU Dresden, Dresden, Germany; 7 Department of Physics, Freie Universität Berlin, Berlin, Germany

## Abstract

We develop a theoretical foundation for a time-series analysis method suitable for revealing the spectrum of diffusion coefficients in mixed Brownian systems, for which no prior knowledge of particle distinction is required. This method is directly relevant for particle tracking in biological systems, in which diffusion processes are often nonuniform. We transform Brownian data onto the logarithmic domain, in which the coefficients for individual modes of diffusion appear as distinct spectral peaks in the probability density. We refer to the method as the logarithmic measure of diffusion, or simply as the logarithmic measure. We provide a general protocol for deriving analytical expressions for the probability densities on the logarithmic domain. The protocol is applicable for any number of spatial dimensions with any number of diffusive states. The analytical form can be fitted to data to reveal multiple diffusive modes. We validate the theoretical distributions and benchmark the accuracy and sensitivity of the method by extracting multimodal diffusion coefficients from two-dimensional Brownian simulations of polydisperse filament bundles. Bundling the filaments allows us to control the system nonuniformity and hence quantify the sensitivity of the method. By exploiting the anisotropy of the simulated filaments, we generalize the logarithmic measure to rotational diffusion. By fitting the analytical forms to simulation data, we confirm the method’s theoretical foundation. An error analysis in the single-mode regime shows that the proposed method is comparable in accuracy to the standard mean-squared displacement approach for evaluating diffusion coefficients. For the case of multimodal diffusion, we compare the logarithmic measure against other, more sophisticated methods, showing that both model selectivity and extraction accuracy are comparable for small data sets. Therefore, we suggest that the logarithmic measure, as a method for multimodal diffusion coefficient extraction, is ideally suited for small data sets, a condition often confronted in the experimental context. Finally, we critically discuss the proposed benefits of the method and its information content.

## Significance

Determining molecular diffusion coefficients is essential for understanding transport in biomolecular systems. There are many biological systems in which molecules display multiple modes of diffusion. For example, multiple molecular species may be mixed or a single species may undergo diffusive state transition. Unfortunately, it is often not possible to label the molecules in a way that directly indicates their diffusive states. Thus, there is a need for novel methods for revealing mixed-mode diffusion coefficients. We introduce a data analysis method for extracting a spectrum of diffusive states directly from molecular trajectory data, for which distinct labeling is not required. The method is general, easy to apply, and is fundamentally grounded on a theoretical foundation.

## Introduction

Single-particle/molecule tracking (SPT/SMT) provides a powerful tool for revealing discrete dynamics in many biological systems. Although there is still room for further development of detection and tracking algorithms, the field of SPT/SMT software is well matured, and comparisons between many methods can be found in the literature (1, 2, 3). Unlike the development of the tracking algorithms themselves, techniques for the analysis of the time-series data generated by these algorithms will remain an area of active development. Owing to the diversity of the fields of interest for SPT/SMT, there will be a long-lasting interest in the development of novel analysis techniques.

One area for which SPT/SMT is ideally suited is in the study of Brownian motion, which is a key transport mechanism in biological cells and many other molecular systems. Often in such systems, we are confronted with nonuniform mixtures of diffusing particles and molecules. The different modes of diffusion present in a sample may be the result of mixing multiple molecular species, or they may be due to the action of diffusive state transitions by a single molecular species. When analyzing nonuniform Brownian data, such as one might obtain using SPT/SMT, it is difficult to discern the distinct diffusive modes in mixed systems unless one can distinctly label the molecules by state or by species. Recently, one of the authors (I.H.) has shown that by first transforming nonuniform Brownian data to the logarithmic domain, one can accurately evaluate the underlying spectra of diffusion coefficients (4, 5, 6, 7). This logarithmic measure of diffusion reveals multiple modes of dynamics without the need for distinct labeling. Applied to the study of DNA transport in the presence of a substrate surface, a logarithmic measure of the distribution of individual molecular diffusion coefficients was key in disentangling the mixed-mode surface absorption versus near-surface diffusion effects (4). By analyzing total internal reflection fluorescence microscopy data, the authors showed that there is a correlation between the temporal length of a molecular trajectory and its resultant diffusion coefficient. Longer-lived trajectories were more likely to interact with the substrate surface and hence transition into a lower diffusive mode. A logarithmic analysis of the distribution of individual DNA molecule diffusion coefficients, decomposed over trajectory lifetimes, quantitatively revealed the distinct, state-dependent diffusive modes. When applied to the Brownian displacements of nanoparticles confined by laser trapping, the logarithmic measure revealed a simultaneous reduction of diffusion due to the trapping and an enhanced motion due to the constant input of scattering forces (5). The distinct diffusive modes were spatially organized in response to the trapping force field and were therefore not species dependent; thus, distinct labeling was not an option. Presented in the linear domain, the nonuniformity would be indistinguishable. The logarithmic measure also proved useful for identifying tracking errors in SPT/SMT algorithms due to the false-linking effects (6), thus minimizing the use of trial and error when selecting appropriate algorithm parameters. Additional examples include the identification of nanoparticle crystallization precursors (8), the characterization of material nonuniformity in dense aqueous cellulose nanofiber dispersions (7), and the characterization of large regions of structural order in dispersed nanoparticle mixtures (9). Taken together, these case studies indicate that the logarithmic measure of diffusion provides a simple but powerful tool for understanding phenomena in which nonuniform diffusion occurs. However, currently there is still no theoretical foundation for this method.

Other methods exist for detecting nonuniformity in SPT trajectories. These include the subtrajectory analysis methods, which locally resolve particle trajectories into transient segments of bound, subdiffusive, diffusive, and superdiffusive behavior (10, 11, 12). These segmentation methods characterize varying complex behavior typical of biological systems and particles embedded in active media (13,14) and employ mean squared displacements (MSDs) to classify dynamic characteristics along a particle trajectory. By necessity, these methods require long trajectories, which are not always accessible. More sophisticated methods include the hidden Markov models (15, 16, 17, 18, 19, 20), which determine the diffusion coefficients for distinct diffusive states and the transition rates between states but often only consider a predefined fixed number of states, and other advanced trajectory segmentation methods (21). Advanced Bayesian methods (22) and combined hidden Markov model-Bayesian methods (20) can learn both the number of diffusive states and the transition rates but require a high degree of computational proficiency on behalf of the user. Finally, one can employ recent deep learning techniques with impressive results (23); however, here neural network training is required, which typically requires access to large training data sets.

The purpose of this work is to provide a rigorous foundation for a method that represents a set of benefits that are unique in comparison to these other methods. In the limit case of monomodal diffusion, our method proves to be as accurate as the MSD-based methods for small data sets, i.e., few trajectories, which we show to be true for both short and long trajectories. For large data sets, the number of distinct diffusive states can be determined without prior knowledge and in many instances does not require any fitting or the implementation of advanced computational analysis tools. More generally, our method can be adapted to provide a model selection for *N*-state diffusion. To implement model selection, one does not require advanced Bayesian methods or machine learning techniques but simply the analytic results provided in this work and a generic fitting algorithm provided by most standard numerical analysis packages. In summary, we provide a theoretical foundation for a Brownian data transformation scheme that is comparable to the MSD approaches in accuracy for evaluating diffusion coefficients and evaluates Brownian multimodality in an easy-to-implement way with results that are comparable with the most advanced and sophisticated methods.

We validate the theoretical foundation presented in this work by comparing it with numerical data generated using Brownian dynamics simulations of gelated filament bundles. There are multiple reasons for choosing this system. The first is that it has direct biological relevance with broad implications in the study of cellular biology and biological soft-matter physics. Bundles of filamentous polymers, including actin, microtubules, and intermediate filaments, are pervasive in biological cells, providing the foundation of the cellular cytoskeleton (24), the actomyosin cortex (25), the machinery responsible for chromosome segregation during cell division (26), and other cellular structures (27). Many of these systems are active, being driven away from equilibrium by a range of molecular motor proteins (28), including kinesin (29,30) and dynein (31,32) in the case of microtubules and myosin (33) in the case of actin. As well as providing mechanical forces to drive cellular processes, molecular motors and other passive protein molecules provide cross-linking tether interactions between filament pairs. These tethering bonds connect filaments into larger networked material structures. The novel active and passive material properties of these filamentous materials have generated much interest in the soft-matter physics community (34, 35, 36, 37). Other novel filamentous materials, such as cellulose nanofibers, are gaining traction in the engineering community for their potential as environmentally sustainable and renewable “wonder materials” (38,39). Thus, by modeling the diffusion of bundled filaments, we are generating data representative of biophysical and engineering systems alike. Furthermore, progression to the study of general diffusivities, such as subdiffusive and superdiffusive processes, is natural in the context of filamentous gels. A second reason for choosing bundled filaments rather than, say, polydisperse suspensions of spherical particles is that because of the intrinsic anisotropy of the filaments, we have access to additional diffusive degrees of freedom, which we can use to further validate our theoretical results. As we will see, the generalized protocol presented here for representing the logarithmic measure of diffusion can reveal nonuniformity in both one-dimensional (1D) rotational diffusion and two-dimensional (2D) translational diffusion, confirming the method’s generality. Finally, we mention that by bundling filaments with cross-linking interactions the nonuniformity in the system arises because of particle interactions, rather than by direct construction. Owing to this additional complexity in our choice of the simulation model, the agreement between theoretical and numerical results is an additional testament to the validity of the former.

The outline of this work is as follows: we first review a collection of relevant methods for calculating diffusion coefficients from SPT/SMT time-series data. We then develop the theoretical foundation for generating the logarithmic measure of diffusion in normal monomodal and bimodal translational diffusion, referring to a generalized protocol included in Appendix A. We then introduce the filament bundle simulation method and compare numerical results with theoretical results. Finally, we apply the methods developed here to the case of the 1D rotational diffusion of the anisotropic filament bundles and discuss the implication of our results for the study of nonuniform diffusion.

## Methods

### Methods for calculating diffusion coefficients

We review several methods of interest for determining diffusion coefficients from SPT/SMT data and discuss their relevance in the context of evaluating diffusive nonuniformity. Note that because the purpose of this work is to establish a theoretical foundation for the logarithmic transformation method, we do not account for measurement errors in this treatment. We address this limitation again in the Conclusions.

The standard method for evaluating diffusion coefficients is to use the mean-squared displacement:

(1) $$D_{\text{MSD}}=\underset{t\to \infty }{\text{lim}}\frac{1}{2n_{\text{d}}Nt}\overset{N}{\underset{i=1}{\sum }}|r_{i}\left(t\right)-r_{i}\left(0\right){|}^{2},$$

where **r**_*i*_(*t*) is the position of the *i*^th^ particle at time *t*, *n*_d_ is the number of spatial dimensions, and *N* is the number of molecules or particles in the sample. *D*_MSD_ is defined in the infinite time limit. However, in practice it must be approximated in finite time. For our discussion, it is important to note that Eq. 1 cannot evaluate polydispersed diffusion coefficients without some prior knowledge of particle states or distinct particle labeling.

Typically, there are two alternative formulas for evaluating finite-data representations of the diffusion coefficient. The first is to calculate a frame-based average coefficient:

(2) $$D_{\text{FB}}=\frac{1}{2n_{\text{d}}\text{Δ}t\sum_{i=1}^{N_{\text{I}}}\left(N_{\text{F}i}-1\right)}\overset{N_{\text{I}}}{\underset{i=1}{\sum }}\overset{N_{\text{F}i}-1}{\underset{j=1}{\sum }}|r_{i}(t_{j+1})-r_{i}(t_{j}){|}^{2}.$$

*N*_I_ is the number of individual detected particles and *N*_F*i*_ is the number of sequential frames over which the *i*^th^ particle is detected. **r**_*i*_(*t*_*j*_) is the position of the *i*^th^ particle in the *j*^th^ frame, with Δ*t* being the time interval between frames. All displacements are evenly weighted so that *D*_FB_ represents the mean of all displacements squared, divided by the time interval. A second approach is to calculate an individual-based average:

(3) $$D_{\text{IB}}=\frac{1}{N_{\text{I}}}\overset{N_{\text{I}}}{\underset{i=1}{\sum }}[\frac{1}{2n_{\text{d}}\text{Δ}t\left(N_{\text{F}i}-1\right)}\overset{N_{\text{F}i}-1}{\underset{j=1}{\sum }}|r_{i}(t_{j+1})-r_{i}(t_{j}){|}^{2}].$$

Here, the mean of the displacements squared is first calculated for each particle, then the average of the means is calculated over all particles. Thus, the contributions from all displacements are not evenly weighted in the final value. In the presence of nonuniformity, Eqs. 2 and 3 will not be equivalent. Comparisons between *D*_FB_ and *D*_IB_ have therefore been used to quantify nonuniformity in systems that exhibit diffusive state transitions (4,40). There is no need for prior knowledge of the diffusive state of a particle. Furthermore, because particle lifetimes can vary, one can analyze *D*_FB_ and *D*_IB_ in subsets of trajectory lengths. Such a decomposition provides additional information about system nonuniformity. For instance, in the context of surface absorption processes detected in a total internal reflection fluorescence microscopy field, particles with longer trajectories are more likely to contribute smaller values to *D*_IB_ because they are more likely to interact with the surface. Such individuality will have less of an impact on *D*_FB_; hence, the two quantities will not, in general, be equal.

A third quantity, which we refer to throughout this work as the diffusion element, is given by

(4) $$S_{ij}=\frac{1}{2n_{\text{d}}\text{Δ}t}|r_{i}(t_{j+1})-r_{i}(t_{j}){|}^{2}.$$

*S*_*ij*_ is not an averaged quantity like the diffusion coefficients discussed above. It is simply the normalized displacement squared per particle per time step. Because we divide by the time element Δ*t*, *S*_*ij*_ has units of diffusion. However, it is not a diffusion coefficient. In fact, *D*_FB_ is the mean of *S*_*ij*_-values. Recently, it was shown that by plotting the distribution of the logarithm of *S*_*ij*_-values, one can clearly distinguish between true particle displacements and displacement artifacts introduced by tracking algorithms (6). It has been proposed that distinct diffusive modes in a Brownian data set can also be revealed as distinct spectral peaks in the distribution of the logarithm of *S*_*ij*_-values, hence revealing nonuniformity. In the following, we provide a theoretical foundation for this proposal and benchmark the results using numerical simulation.

### The logarithmic measure of diffusion

We start by developing an analytic expression for the logarithmic measure of the diffusion of a monomodal population of Brownian particles in 2D. We follow the steps of the generalized procedure outlined in Appendix A. Then, we provide an analytic expression for the logarithmic measure of diffusion for a 2D bimodal mixture of Brownian particles.

#### Monomodal system

Brownian displacements in 2D are given by two independent and identically distributed (IID) random variables *dx* and *dy*, with Gaussian probability densities of zero mean and variance *σ*^2^:

(5) $$\begin{array}{l} f\left(dx;\sigma \right)=\frac{1}{\sigma \sqrt{2\pi }}\text{exp}\left[-\frac{dx^{2}}{2\sigma^{2}}\right],\\ f\left(dy;\sigma \right)=\frac{1}{\sigma \sqrt{2\pi }}\text{exp}\left[-\frac{dy^{2}}{2\sigma^{2}}\right]. \end{array}$$

We introduce a new variable, *Z* = *dx*^2^ + *dy*^2^, which is a bivariate transformation of the displacements. The corresponding diffusion element in 2D is then

(6) $$S\left(dx,dy\right)=\frac{dx^{2}+dy^{2}}{4\text{Δ}t},$$

or simply *S* = *Z*/(4Δ*t*). Our aim is to find the probability density of the quantity log_10_[*S*(*dx*, *dy*)].

Following step 3 in Appendix A, an initial pair of random variable transformations map *dx* → *X* and *dy* → *Y* according to *g*_*X*_(*dx*) = *dx*^2^ = *X* and *g*_*Y*_(*dy*) = *dy*^2^ = *Y*, where *g* indicates a transformation, with inverse transformations $g_{X,\pm }^{-1}=\pm \sqrt{X}$ and $g_{Y,\pm }^{-1}=\pm \sqrt{Y}$ and derivatives $dg_{X,\pm }^{-1}/dX=\pm 1/\left(2\sqrt{X}\right)$ and $dg_{Y,\pm }^{-1}/dY=\pm 1/\left(2\sqrt{Y}\right)$. The transformations of the probability densities *f*(*dx*; *σ*) → *F*(*X*; *σ*) and *f*(*dy*; *σ*) → *F*(*Y*; *σ*) are therefore given by

(7) $$\begin{array}{l} F\left(X;\sigma \right)=\underset{i=+,-}{\sum }f\left(g_{X,i}^{-1};\sigma \right)\left|\frac{dg_{X,i}^{-1}}{dX}\right|=\frac{1}{\sigma \sqrt{2\pi X}}\text{exp}\left[-\frac{X}{2\sigma^{2}}\right]\\ F\left(Y;\sigma \right)=\underset{i=+,-}{\sum }f\left(g_{Y,i}^{-1};\sigma \right)\left|\frac{dg_{Y,i}^{-1}}{dY}\right|=\frac{1}{\sigma \sqrt{2\pi Y}}\text{exp}\left[-\frac{Y}{2\sigma^{2}}\right]. \end{array}$$

*F* is *χ*^2^ distributed with one degree of freedom. We write *F* as a *χ*^2^ distribution in terms of gamma functions:

(8) $$\begin{array}{l} F\left(X;\sigma \right)=\frac{1}{{\left(2\sigma^{2}\right)}^{\frac{1}{2}}}\frac{X^{\frac{1}{2}-1}}{\text{Γ}\left(\frac{1}{2}\right)}\text{exp}\left[-\frac{X}{2\sigma^{2}}\right]\\ F\left(Y;\sigma \right)=\frac{1}{{\left(2\sigma^{2}\right)}^{\frac{1}{2}}}\frac{Y^{\frac{1}{2}-1}}{\text{Γ}\left(\frac{1}{2}\right)}\text{exp}\left[-\frac{Y}{2\sigma^{2}}\right], \end{array}$$

where by identity $\text{Γ}\left(1/2\right)=\sqrt{\pi }$. Because of the additivity of *χ*^2^ distributions, the probability density of *Z* = *X* + *Y* is also *χ*^2^ distributed with two degrees of freedom:

(9) $$F\left(Z;\sigma \right)=\frac{1}{2\sigma^{2}}\text{exp}\left[-\frac{Z}{2\sigma^{2}}\right],$$

where we have used the identity Γ(1) = 1.

We introduce the new variable *η* = log_10_(*S*) and perform a second transformation *Z* → *η*, such that *g*_*η*_(*Z*) = log_10_(*Z*) − log_10_(4Δ*t*) = *η*, with transformation inverse $g_{\eta }^{-1}$ = 4Δ*t*10^*η*^ and derivative $dg_{\eta }^{-1}$/*dη* = 4Δ*t*ln(10)10^*η*^. Finally, we transform the probability density *F*(*Z*; *σ*) → *H*(*η*; *σ*) such that

(10) $$H\left(\eta ;\sigma \right)=F\left(g_{\eta }^{-1};\sigma \right)\left|\frac{dg_{\eta }^{-1}}{d\eta }\right|=\lambda 10^{\eta }\text{exp}\left[-\frac{2\text{Δ}t10^{\eta }}{\sigma^{2}}\right],$$

where *λ* = 2ln(10)Δ*t*/*σ*^2^. *H*(*η*)is the distribution of the logarithmic measure of diffusion elements *S* given by Eq. 6. The so-defined diffusion coefficient *D*_S_ follows the normal relationship with the variance *σ*^2^ = 2*D*_S_Δ*t*.

#### Bimodal mixture

The displacement variables *dx* and *dy* and the number of degrees of freedom are the same as in the monomodal system. However, from the generic form for the probability densities given in Eq. A1, the densities for the bimodal mixtures are

(11) $$\begin{array}{l} f^{\text{bi}}\left(dx;\sigma_{1},\sigma_{2}\right)=\alpha f\left(dx;\sigma_{1}\right)+\left(1-\alpha \right)f\left(dx;\sigma_{2}\right)\\ f^{\text{bi}}\left(dy;\sigma_{1},\sigma_{2}\right)=\alpha f\left(dy;\sigma_{1}\right)+\left(1-\alpha \right)f\left(dy;\sigma_{2}\right). \end{array}$$

Here, *f*(*dx*; *σ*_*k*_) and *f*(*dy*; *σ*_*k*_) are given by Eq. 5, with distinct modal variances $\sigma_{1}^{2}$ and $\sigma_{2}^{2}$. *α* gives the proportion of mixing for a binary system. *S* is given by Eq. 6. Following the procedure outlined above, we find that the probability density for *Z* in a bimodal mixture

(12) $$F\left(Z;\sigma_{1},\sigma_{2}\right)=\frac{\alpha }{2\sigma_{1}^{2}}\text{exp}\left[-\frac{Z}{2\sigma_{1}^{2}}\right]+\frac{1-\alpha }{2\sigma_{2}^{2}}\text{exp}\left[-\frac{Z}{2\sigma_{2}^{2}}\right].$$

Subsequently, we generate the logarithmic measure:

(13) $$H\left(\eta ;\sigma_{1},\sigma_{2}\right)=\alpha \lambda_{1}10^{\eta }\text{exp}\left[-\frac{2\text{Δ}t10^{\eta }}{\sigma_{1}^{2}}\right]+\left(1-\alpha \right)\lambda_{2}10^{\eta }\text{exp}\left[-\frac{2\text{Δ}t10^{\eta }}{\sigma_{2}^{2}}\right],$$

where *λ*_1_ = 2ln(10)Δ*t*/$\sigma_{1}^{2}$ and *λ*_2_ = 2ln(10)Δ*t*/$\sigma_{2}^{2}$. Again, we can relate the diffusion coefficients to the variances: $\sigma_{1}^{2}$ = 2*D*_*S*1_Δ*t* and $\sigma_{2}^{2}$ = 2*D*_*S*2_Δ*t*.

### Brownian simulation of filament bundles

All of the simulation data presented in this work are generated using 2D Brownian dynamics simulations of permanently cross-linked rigid-filament colloids, which we refer to as gelated filament bundles. In this section, we present the technical details of the simulation method. Filaments are described by a center-of-mass position vector **r**_*i*_(*t*) and an orientation unit vector ${\hat{u}}_{i}\left(t\right)$, where *i* = 1, 2, …, *N*, and *N* is the total number of filaments. All filaments have constant length *L* and diameter *d* and exhibit anisotropic drag with three drag coefficients given by (41)

(14) $$\gamma_{∥}=\frac{2\pi \zeta L}{\text{ln}\left(L/d\right)},\;\gamma_{⊥}=\frac{4\pi \zeta L}{\text{ln}\left(L/d\right)},\;\gamma_{\text{r}}=\frac{\pi \zeta L^{3}}{3\text{ln}\left(L/d\right)},$$

where *ζ* is the solvent viscosity. The coefficients *γ*_||_ and $\gamma_{⊥}$ are for drag parallel and perpendicular to the filament long axis, respectively, and *γ*_r_ is the coefficient for rotational drag. Correspondingly, we can define three diffusion coefficients *D*_||_ = *k*_B_*T*/*γ*_||_, $D_{⊥}=k_{\text{B}}T/\gamma_{⊥}$, and *D*_r_ = *k*_B_*T*/*γ*_r_, where *k*_B_ is the Boltzmann constant and *T* is the absolute temperature. In 2D, the diffusion coefficient for the total center of mass will be *D* = (*D*_||_ + $D_{⊥}$)/2.

The equations of motion describing filament dynamics are

(15) $$\begin{array}{l} dr_{i}=\Gamma_{i}^{-1}\cdot \left[\overset{N}{\underset{j\neq i}{\sum }}F_{ij}^{\phi }+\overset{N}{\underset{j\neq i}{\sum }}\overset{N_{\alpha }}{\underset{\alpha =1}{\sum }}F_{ij,\alpha }^{\text{cl}}\right]dt+\delta r_{i}^{\text{B}}\\ d{\hat{u}}_{i}=\frac{1}{\gamma_{\text{r}}}\left[\overset{N}{\underset{j\neq i}{\sum }}\left(F_{ij}^{\phi }\times \lambda_{ij}{\hat{u}}_{i}\right)+\overset{N}{\underset{j\neq i}{\sum }}\overset{N_{\alpha }}{\underset{\alpha =1}{\sum }}\left(F_{ij,\alpha }^{\text{cl}}\times \varepsilon_{ij,\alpha }{\hat{u}}_{i}\right)\right]dt\times {\hat{u}}_{i}+\delta {\hat{u}}_{i}^{\text{B}}. \end{array}$$

Here, $\delta r_{i}^{\text{B}}$ and $\delta {\hat{u}}_{i}^{\text{B}}$ are the Brownian displacements and rotations, respectively (42). These terms satisfy the fluctuation-dissipation relation such that $\langle \delta r_{i}^{\text{B}}\left(t\right)\delta r_{i}^{\text{B}}\left(t^{\prime }\right)\rangle =2k_{\text{B}}T\Gamma_{i}^{-1}\delta \left(t-t^{\prime }\right)dt$ and $\langle \delta {\hat{u}}_{i}^{\text{B}}\left(t\right)\delta {\hat{u}}_{i}^{\text{B}}\left(t^{\prime }\right)\rangle =2k_{\text{B}}T\left(I-{\hat{u}}_{i}{\hat{u}}_{i}\right)\delta \left(t-t^{\prime }\right)dt/\gamma_{i}^{\text{r}}$, where **Γ**_*i*_ = $\gamma_{⊥}{\hat{u}}_{i}{\hat{u}}_{i}$ + *γ*_||_(**I** − ${\hat{u}}_{i}{\hat{u}}_{i}$) is the anisotropic friction tensor and **I** is the identity matrix. $F_{ij}^{\phi }$ is the purely repulsive steric interaction force between two filaments *i* and *j*, calculated along the line of shortest interaction between the two filaments. The contact locations for pairwise steric interaction forces between two filaments are given by a pair of line parameters: −*L*_*i*_/2 ≤ *λ*_*ij*_ ≤ *L*_*i*_/2 for the point of contact on filament *i* due to an interaction with filament *j* and −*L*_*j*_/2 ≤ *λ*_*ji*_ ≤ *L*_*j*_/2 for the point of contact on filament *j* due to an interaction with filament *i*. The direction of a steric force is given by the vector connecting the two points of contact, and the magnitude is given by the standard Weeks-Chandler-Andersen (WCA) interaction (43), where the Lennard-Jones distance is given by the filament diameter and the energy is equal to 1*k*_B_*T*.

**Figure 1:**
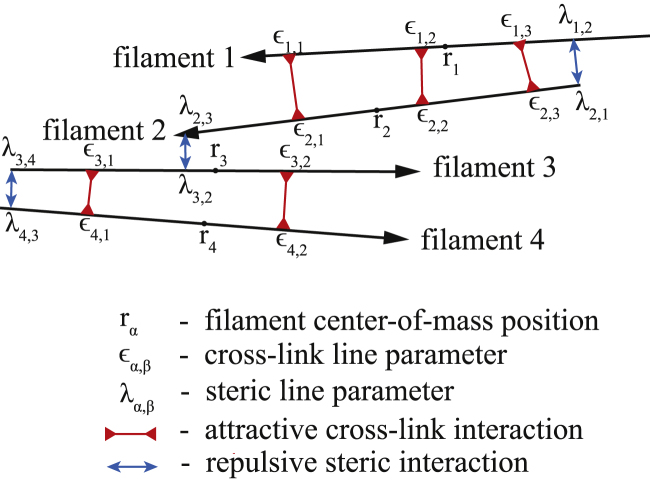
A schematic representation of filament interactions. Two cross-linked filament bundles of size 2 are shown to be interacting via pairwise steric interactions and cross-linking bonds. We indicate all interaction line parameters. To see this figure in color, go online.

$F_{ij,\alpha }^{\text{cl}}$ is a permanent tether bond cross-linking two filaments *i* and *j*. For each cross-linked filament pair, there are *N*_*α*_ bonds, indexed by *α*. The sites for the *α*^th^ tethering contact between filaments *i* and *j* are given by a pair of line parameters: −*L*_*i*_/2 ≤ *ɛ*_*ij*,*α*_ ≤ *L*_*i*_/2 and −*L*_*j*_/2 ≤ *ɛ*_*ji*,*α*_ ≤ *L*_*j*_/2 (44). The direction of a bond force is taken from the vector between the two bond sites. The magnitude of the force is calculated using a spring potential $u(d_{ij})=\frac{1}{2}\kappa {\left(d_{ij}\;-\;d_{0}\right)}^{2}$, where *d*_*ij*_ is the distance between the two binding sites, *d*_0_ is the equilibrium spring length, and *κ* is the stiffness of the spring. A schematic representation of the filament interactions is shown in Fig. 1.

**Figure 2:**
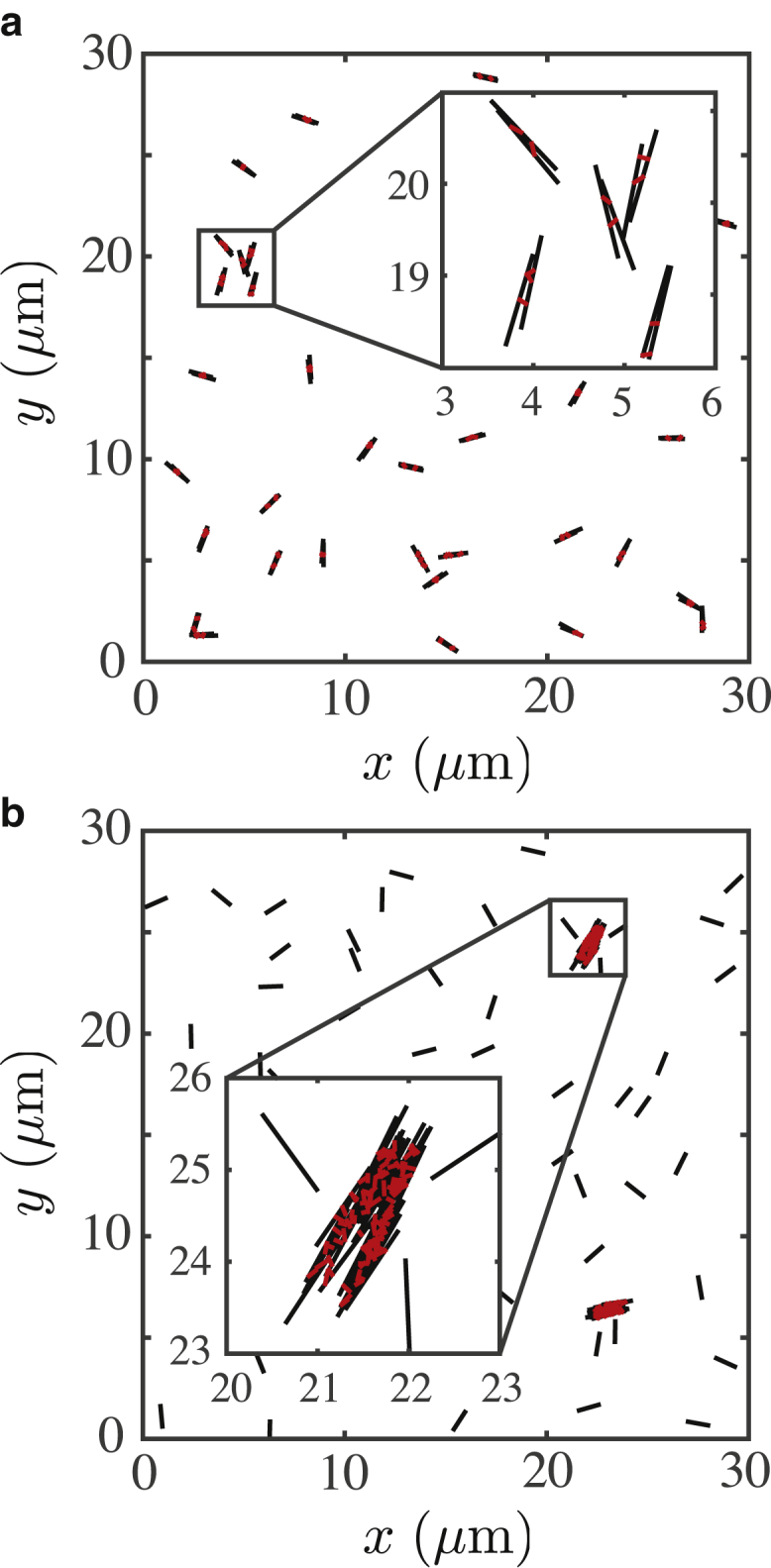
Brownian dynamics simulation of cross-linked filament bundles in 2D. Filaments are shown in black, and cross-linking bonds are shown in red. (*a*) A monodispersed, uniform system with bundles of size 2 is shown. (*b*) A bimodal, nonuniform mixture containing bundles of size 30 interacting with single, unbound filaments is shown. To see this figure in color, go online.

**Figure 3:**
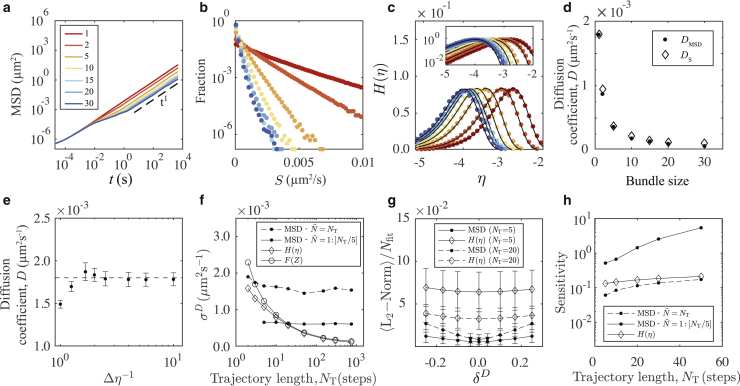
Analysis of monomodal systems. (*a*) MSD for a range of filament bundle sizes is shown. The large-timescale diffusive regime is indicated with a dashed line that is linear in *t*. The color scheme for the bundle sizes, indicated in the legend, is used throughout this figure. (*b*) Normalized histograms for the distribution of *S* plotted on a log-linear scale are shown. (*c*) The same data as in (*b*) are shown with histograms accumulated over the *η* domain. The black lines are fits using Eq. 10. The log inset shows the distribution peaks, which we use to evaluate the diffusion coefficients. (*d*) Comparison of the diffusion coefficients calculated using the MSD in (*a*) and the fit of the logarithmic measure Eq. 10 in (*c*) is given. (*e*) Histogram bin width dependence for fitting Eq. 10 (bundle size 1) is shown. The dashed line is the ground truth for a single filament given by Eq. 14. (*f*) The SD for single-filament diffusion coefficients calculated by three different methods as a function of trajectory length (*N*_T_) is shown. Sample size *N*_s_ = 3000 trajectories. The variance is calculated against the ground truth. (*g*) Fitting robustness is expressed as the mean of the L_2_-norm per fitting data point $\langle {\text{L}}_{2}-\text{norm}\rangle$/*N*_fit_, calculated for each filament with *N*_T_ = 5, 20. *δ*^*D*^ is the variation imposed on the diffusion coefficient for the calculation of residuals in the norm such that *δ*^*D*^ = 0 indicates the optimal fit L_2_-norm. Error bars indicate the SD of the L_2_-norm over the set of individual filaments. (*h*) Sensitivity is given, represented as the curvature of the robustness curves in (*g*), i.e., the second derivative at *δ*^*D*^ = 0. To see this figure in color, go online.

All simulations are performed in 2D squares with periodic boundary conditions. The equations of motion in Eq. 15 are numerically approximated using a forward Euler time integration scheme with a time step size of 5 *μ*s. Particle tracking follows the filament center-of-mass trajectories, which in 2D are given by **r**_*i*_(*t*) = (*x*_*i*_(*t*), *y*_*i*_(*t*)). The time increment between successive points in the time-series analysis, Δ*t*, is generally greater than the simulation time step, except in the calculation of mean-square displacements in Fig. 3 *a*. For each set of parameters, we run 50 independent simulations, each running for 2000 s of simulated time, such that the total simulation time per parameter set is 1 × 10^5^ s. The constant length and diameter of the filaments are *L* = 1 *μ*m and *d* = 25 nm, respectively. For all simulations, *T* = 300 K and *ζ* = 1 Pa ⋅ s. For the cross-linker interactions, we use a spring stiffness of *κ* = 0.3 pN/nm and an equilibrium length of *d*_0_ = 80 nm, which are parameters representative of an Eg-5 kinesin molecular motor (28). All cross-link bonds are formed as an initial condition. Subsequently, no bond is either created or destroyed throughout the simulation. We use bundle sizes of 1, 2, 5, 10, 15, 20, and 30 filaments. Monodispersed systems contain bundles of only one size. We show an example of a monodispersed system in Fig. 2 *a*. Permanent bundles of two filaments are held together by cross-linking bonds. Filaments will diffuse and interact according to Eq. 15 and Fig. 1. The inset shows a detailed magnification of a small group of bundles. Note that the bundles are in general sparsely distributed owing to the low overall filament concentration. For higher concentrations, steric interactions become more frequent, and filaments exhibit subdiffusive dynamics. In the high concentration limit, systems exhibit caging effects and jamming. We maintain a low concentration throughout to avoid these high-density effects. For binary mixtures, we mix bundles of size 2, 5, 10, 15, 20, and 30 with populations of single, unbound filaments. We show an example of a binary mixture in Fig. 2 *b*. Here, we have two bundles, each containing 30 filaments, surrounded by a population of freely diffusing, unbound single filaments. The inset shows a magnification of an example bundle structure. All simulations are performed in 2D periodic squares with dimensions *L*_*x*_ × *L*_*y*_ = 30 *μ*m × 30 *μ*m. Typically, we use a total of 120 filaments per simulation, except for the case of investigating variations in mixing parameter *α*.

All simulations were performed using custom software implemented in C++, and analysis was performed using custom MATLAB (The MathWorks, Natick, MA) scripts. Simulation software can be downloaded by following this link: https://mosaic.mpi-cbg.de/?q=downloads/filament_bundles.

## Results and discussion

To establish the validity of the theoretical results developed in The logarithmic measure of diffusion, we begin by considering monomodal systems. We simulate cross-linked filament bundles and calculate collective bundle diffusion coefficients as a function of bundle size. We compare results obtained using Eq. 10 with results calculated using the MSD given by Eq. 1. Fig. 3 *a* shows the MSD calculated over nine decades of time for bundles of size 1, 2, 5, 10, 15, 20, and 30. There appear to be three dynamic regimes: an approximately diffusive regime for timescales less than 10^−2^ s, a subdiffusive regime between 10^−2^ s and 10^0^ s, and a second diffusive regime for larger timescales. The subdiffusive regime dominates as the filaments sample the harmonic potential introduced by the cross-linking bond interactions. The contribution of subdiffusive dynamics becomes more pronounced for larger bundle sizes. This is because the filaments near the center of a cluster experience erratic increases in confinement because of fluctuations in cluster filament density. At shorter timescales, the filaments explore, on average, the region of the potential where the bond force is insignificant compared with thermal forces. Varying the spring stiffness parameter *κ* will vary the transition timescale between these two dynamic regimes. At larger timescales, we see the collective diffusion at which individual bundles migrate as a whole. To evaluate the collective diffusion coefficient of a bundle, we therefore consider only the MSD on large timescales. This region is indicated by the t^1^ dashed line in Fig. 3 *a*. For each of the bundles, we fit a linear function to this region of the MSD and calculate the effective bundle diffusion coefficient from the slope. The results for the collective diffusion coefficients *D*_MSD_ calculated in this way are shown in Fig. 3 *d*. Although we have analytic expressions for a single, unbound filament (see Eq. 14), no such expressions are available for bundles. Therefore, we use the diffusion coefficients calculated using the MSD as a baseline for comparison throughout this work.

Next, we investigate the validity of Eq. 10 for monomodal systems. In Fig. 3 *b*, we show the distributions of *S*-values for the same data used to calculate *D*_MSD_. The distributions are calculated by accumulating normalized histograms. We use time increments Δ*t* = 2.5 s. This is a reliable step size for probing the larger timescales of collective bundle diffusion and avoiding the effects of the bond interactions. Plotting the distributions of *S* on a logarithmic scale reveals an almost exponential behavior for all bundle sizes, as is expected from Eq. 9. In principle, one could directly fit these data with the appropriate functional form to reveal the variance and hence the diffusion coefficient. Alternatively, instead of plotting the distribution of *S*, we can plot the distribution of *η* = Log_10_(*S*). Then *η* becomes the independent variable, representing a two-step transformation from the original displacement data. Thus, we accumulate data into histograms with evenly spaced bins on the domain of *η*, with the histogram bin width here Δ*η* = 0.1. The logarithmic measure of diffusion, calculated in this way, is plotted in Fig. 3 *c*. The colored points represent the histogram data for different bundle sizes. We compare the data with our theoretical result by fitting the functional form of Eq. 10 to the histogram data. The solid lines in Fig. 3 *c* show the results of the fitting. The peaks are emphasized in the log inset. The analytic form of Eq. 10 allows us to directly quantify the diffusion coefficients from the histograms. Therefore, we solve for the variance and calculate *D*_S_ = *σ*^2^/2Δ*t*. In Fig. 3 *d*, we plot these values for the diffusion coefficients extracted by fitting the logarithmic measure and compare against the values obtained using the MSD. For the monodispersed system, the two methods are in agreement, confirming that Eq. 10 is an appropriate functional form for the logarithmic measure of monomodal diffusion providing a complementary method for evaluating monomodal diffusion coefficients. As has recently been shown (6), the location of a peak *η*_p_ directly coincides with the diffusion coefficient of a diffusive mode. Visual inspection of *H*(*η*)can therefore reveal the underlying diffusion coefficient because *D*_S_ = $10^{\eta_{\text{p}}}$. This means that in practice, a full fit is not necessary.

We compare the errors associated with the application of Eq. 10 and the MSD by considering only the simplest case of unbound filaments, i.e., a bundle size of 1. In Fig. 3 *e*, we show the dependence of the diffusion coefficient extracted by fitting Eq. 10 as a function of the histogram bin width. Bin widths less than Δ*η* = 0.5 are suitable. We are interested in the accuracy of the different methods depending on track length. In Fig. 3 *f*, we show standard deviations (SDs) for the distributions of diffusion coefficients calculated for individual filaments as a function of trajectory length (*N*_T_). Note that the method for evaluating errors in the diffusion coefficient via the MSD is different from Qian et al. (45). Here, for each MSD associated with an individual filament trajectory, we use linear regression over some selected subrange of the MSD. We use the gradient of the linear fit to calculate the single-filament diffusion coefficient. We determine a mean and SD from the distribution of diffusion coefficients calculated in this way over a population of *N*_s_ = 3000 trajectories. We use internal averaging for each MSD such that for a single trajectory, all displacements over a given time lag contribute to the MSD (3,45). In Appendix B, we discuss the dependence of the errors for the MSD approach on the choice of MSD filtering for the linear fit. If $\tilde{N}$ is the maximal time lag used for filtering the MSD, where $\tilde{N}$ ≤ *N*_T_, then we show that the scaling of the errors, as a function of trajectory length, depends on our choice of $\tilde{N}$. In Fig. 3 *f*, we show two such selections. Naively, one can set $\tilde{N}$ = *N*_T_ and use the full MSD without filtering. Alternatively, one can use a fraction of the full MSD—for example, the first 20%—such that $\tilde{N}$ = *N*_T_/5. The former represents a lower bound on the quality of the errors. The latter represents a more practical choice. In both instances, we see that the error in the MSD approach is constant as a function of trajectory length. In the case of the *H*(*η*) method, the errors scale proportionally to 1/$\sqrt{N_{\text{T}}}$, with no filtering required. In Fig. 8 of Appendix B, we show that for some choices of $\tilde{N}$, the errors associated with the MSD also approach 1/$\sqrt{N_{\text{T}}}$. Additionally, in Fig. 3 *f*, we show that by directly fitting Eq. 12 for *F*(*Z*), we achieve similar results as the *H*(*η*) method.

We also consider the fitting sensitivity of the MSD and *H*(*η*). For the optimal fit of the MSD and *H*(*η*), we calculate the L_2_-norm using the root mean squared (RMS) residual. To evaluate fitting robustness, we impose a variation *δ*^*D*^ on the optimal diffusion coefficient *D*_0_ and recalculate residuals with *D*_res_ = *D*_0_(1 + *δ*^*D*^). In Fig. 3 *g*, we show the means and SDs for the distributions of L_2_-norms calculated over the population of *N*_s_ = 3000 filaments as a function of *δ*^*D*^. For the MSD approach, we show results for $\tilde{N}$ = *N*_T_/5. We assume a quadratic dependence on *δ*^*D*^ for small variations and hence define the fitting sensitivity from the curvature of the curves in Fig. 3 *g*, which we quantify using second derivatives. In Fig. 3 *h*, we show the fitting sensitivity over a range of track lengths for *H*(*η*) and the two implementations of the MSD approach ($\tilde{N}$ = *N*_T_ and *N*_T_/5). With appropriate filtering, the MSD is more sensitive to variations in fitting parameters. However, the *H*(*η*) method outperforms the naive implementations of the MSD. Overall, our results suggest that the accuracy of the *H*(*η*) method is comparable to the various methods that employ the MSD for evaluating diffusion coefficients. There are, however, many ways to filter and extract diffusion coefficients from the MSD (discussed in Appendix B), such that the relative accuracy between the MSD and *H*(*η*) methods depend strongly on this choice. In the case of the *H*(*η*) method, there is little ambiguity in the implementation.

**Figure 4:**
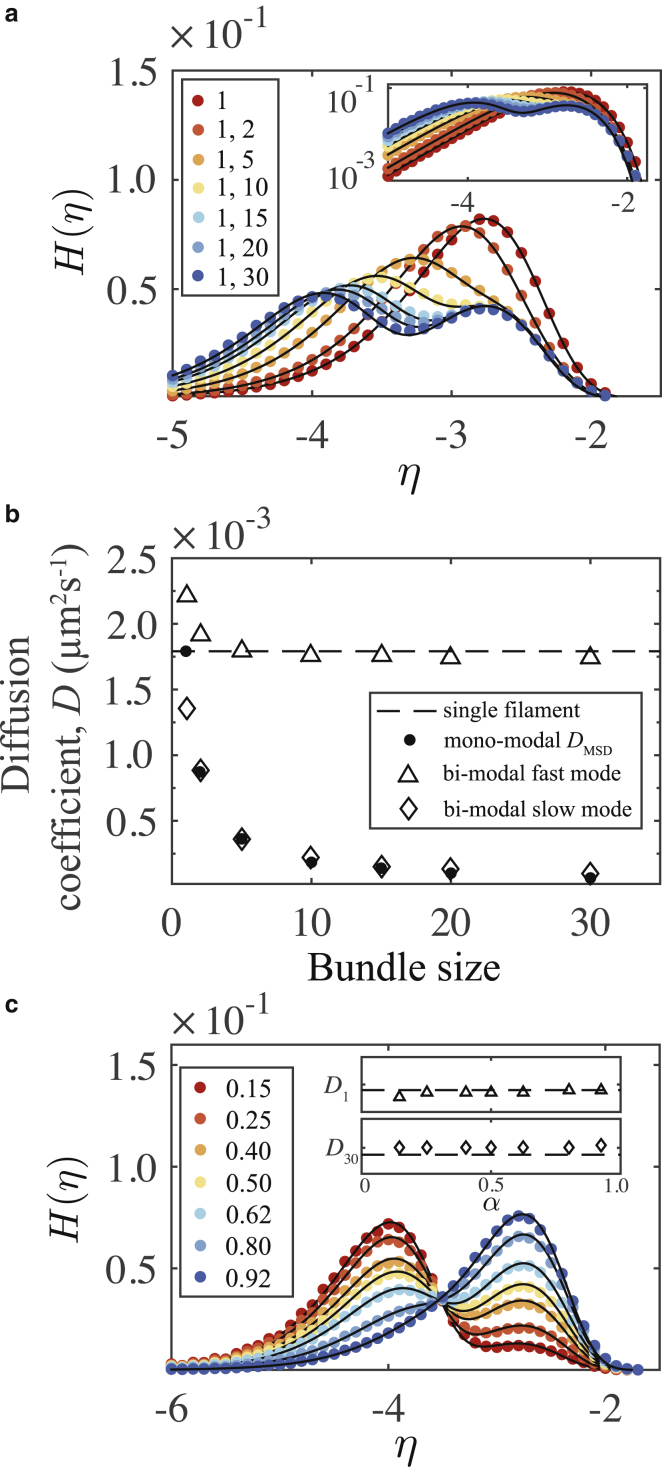
(*a*) Normalized histograms and fits of Eq. 13 for the logarithmic measure of bimodal bundle mixtures. For comparison, data are also shown for the uniform system of free filaments. The figure legend indicates the bundle sizes in the binary mixtures. (*b*) The bimodal diffusion coefficients as extracted by fitting the logarithmic measure are given. Slow modes are compared with values obtained via the MSD in Fig. 3. Fast modes are compared with the uniform system of free filaments. Both the slow mode and the fast mode for each pair are plotted using the slow-mode bundle size as the independent variable. (*c*) Bimodal analysis with fixed modes over a range of *α* is shown. The color legend indicates *α*. The insets show the diffusion coefficients extracted by fitting Eq. 13 to the histogram data compared with the MSD values. To see this figure in color, go online.

More importantly, the approach of extracting diffusion coefficients using the logarithmic measure can be extended to nonuniform systems. Having established that the dynamics of individual filaments depend on the size of the bound network to which they contribute, we now mix bundles of controlled sizes to create Brownian systems with multiple diffusive modes. In Fig. 2 *b*, we show an example of a bimodal system with one population of bundled filaments and a second population of unbound filaments. In Fig. 4 *a*, we show the distributions of *η* for six example binary mixtures. We consider bundles of size 2, 5, 10, 15, 20, and 30, and in each case, we create a binary mixture by adding a population of single unbound filaments. For all systems, we use a total of 60 bound filaments and 60 unbound filaments. Therefore, the mixing ratio is *α* = 1/2. The colored points are for the normalized histograms of *η*, and the solid lines show fits of Eq. 13 fitted to the histogram data. For bundle sizes 2 and 5, it appears to be difficult to distinguish multiple peaks in the distributions. An analysis of the second derivative of Eq. 13

(16) $$\begin{array}{c} \frac{d^{2}H\left(\eta ;\sigma_{1},\sigma_{2}\right)}{d\eta^{2}}=\frac{6\alpha \text{Δ}t}{\sigma_{1}^{2}}\text{ln}\left(10\right)10^{\eta }\left(\frac{4\text{Δ}t^{2}10^{2\eta }}{\sigma_{1}^{4}}-\frac{6\text{Δ}t10^{\eta }}{\sigma_{1}^{2}}+1\right)\text{exp}\left[-\frac{2\text{Δ}t10^{\eta }}{\sigma_{1}^{2}}\right]\\ \;+\frac{6\left(1-\alpha \right)\text{Δ}t}{\sigma_{2}^{2}}\text{ln}\left(10\right)10^{\eta }\left(\frac{4\text{Δ}t^{2}10^{2\eta }}{\sigma_{2}^{4}}-\frac{6\text{Δ}t10^{\eta }}{\sigma_{2}^{2}}+1\right)\text{exp}\left[-\frac{2\text{Δ}t10^{\eta }}{\sigma_{2}^{2}}\right] \end{array}$$

reveals a critical separation of variances at which an additional mode appears, indicating the onset of an inflection point in the bimodal form of *H*(*η*). For all larger differences, there are two roots. Analyzing Eq. 16 with *α* = 1/2 and a fixed fast mode with *σ*_1_ = 0.096 (corresponding to a single-filament diffusion coefficient *D*_S1_ = 1.8 × 10^−3^ *μ*m^2^ s^−1^), we find that an additional mode emerges with an inflection point at approximately *η* = −2.73 for *σ*_2_ = 0.048 (corresponding to *D*_S2_ = 4.6 × 10^−4^ *μ*m^2^ s^−1^). This can be clearly seen in the figure as the emergence of a second distinct peak as we increase the size of the slower-mode bundle. In Fig. 4 *b*, we compare the values of the diffusion coefficients extracted by fitting Eq. 13 to the monomodal MSD values. Even with the minimal separation of dynamics, we are able to accurately extract the diffusion coefficient of the slower mode. For all other mixtures, we obtain an accurate evaluation of both the faster and slower modes. These results confirm that Eq. 13 is an appropriate functional form for the logarithmic measure of normal bimodal diffusion in 2D. Furthermore, we are able to support the proposal that, when transformed to the *η* domain, the distribution of SPT displacement data clearly reveals multiple modes of diffusion, which can be simply discerned by locating the spectral peaks. This is not possible for small separations of modes because the functional inflection point vanishes in the region between the two corresponding peak *η*-values. However, in most experimental contexts, the separation of modes is typically much greater than this (5,6).

To further test the ability of our method for detecting binary modes, we vary the relative population size in a mixture while keeping the modes constant. We fix *D*_S1_ = 1.8 × 10^−3^ *μ*m^2^ s^−1^ and *D*_S2_ = 6.5 × 10^−5^ *μ*m^2^ s^−1^ by mixing a single bundle of size 30 with unbound filaments. We vary *α* by varying the population size of the unbound filaments. In Fig. 4 *c*, we show normalized histograms on the *η* domain and the corresponding fits of Eq. 13. The distinct modes of diffusion are observable over a wide range of *α*. We analyze the inflection points in Eq. 13 and find a critical value of approximately *α* = 0.925. For higher values of *α*, the inflection point associated with the two peaks vanishes, corresponding to a saturation of the mass fraction of the unbound filaments. The insets show the value of the diffusion coefficients extracted by fitting the data. We compare the extracted values with the monomodal value for each cluster size and find the fits to be robust over the entire range of *α*. We note that for the slow mode, extracting the peak by visual inspection is slightly inaccurate. However, the simple approach of associating peaks with diffusion coefficients is still practical and insightful.

So far, we have generated nonuniformity by controlling the size of bundles and their mixing proportions. This approach is representative of many experimental contexts in which various species of molecules are mixed or when the dynamics of a single species disperses due to environmental interactions, such as spatial dispersion in the environment of a focused laser trap (5). The distinct modes indicate the elementary dynamics. When fitting the profiles, the mixing proportion of *α* can be used to find relative concentrations. In systems in which a single species exhibits dynamic diffusive state transitions, the same analysis of *α* gives an indication of the transition rates. However, one must be careful to either ensure that Δ*t* is greater than the characteristic state transition times or to modify the displacement densities to include transient behavior. In this way, our method can provide an alternative to the hidden Markov model analysis methods (15, 16, 17, 18, 19), which capture diffusive state transitions and the corresponding transition state rates.

**Figure 5:**
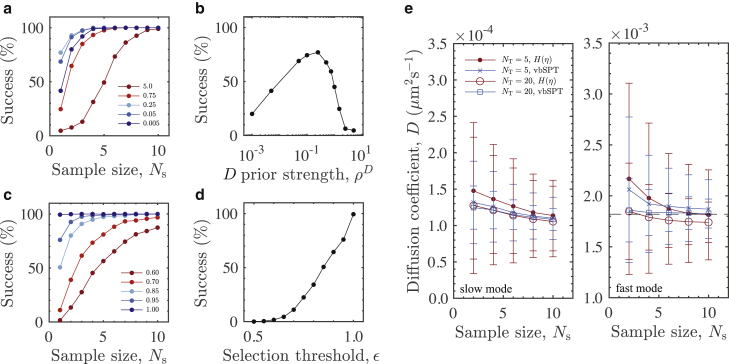
Comparison with the vbSPT method. (*a*) Bimodal detection success for the vbSPT method is shown over a range of sample sizes and diffusion coefficient prior strengths *ρ*^*D*^. Figure legend indicates *ρ*^*D*^. An individual sample is defined as a pair of trajectories containing one free filament and one bundle-embedded filament. For (*a*)–(*d*), all samples have a track length of five steps. The vbSPT method selects an N-state diffusion model using variational Bayesian techniques. The detection power is fully automated and is controlled with the parameter *ρ*^*D*^. (*b*) Bimodal detection success for the vbSPT method for a population of sample size *N*_s_ = 1 is shown as a function of *ρ*^*D*^. The nonmonotonicity indicates an upper limit on the bimodal detection power in the small sample limit. (*c*) Percentage of bimodal detection when selecting between fits of Eqs. 10 and 13 on the logarithmic domain is shown (same data as in *a*). Varying the threshold parameter *ϵ* imposes selection filtering. Figure legend indicates *ϵ*. For *ϵ* = 1 (no filtering), two-state selection is ∼100% successful for all sample sizes. This is clear in (*d*) for a sample size *N*_s_ = 1, which monotonically increases from 0% to ∼100% as *ϵ* → 1. (*e*) compares the accuracy of diffusion coefficients extracted for two modes. Two example trajectory lengths are shown. For a given *N*_s_, the diffusion coefficients are extracted over 1500 independent repetitions of that sample size. Mean and SD (*error bars*) are displayed for the slow and fast modes over a range of sample sizes. Dashed line indicates the ground truth for the fast mode (Eq. 14). To see this figure in color, go online.

To benchmark our method, we compare against the sophisticated variational Bayesian SPT analysis method (vbSPT) of Personn et al. (20). The vbSPT is capable of learning both the number of distinct diffusive states in Brownian data as well as the transition rates for particles that transition between states. We consider the case of a binary mixture with *α* = 0.5 containing free filaments and bundles of size 30. We wish to compare the model selection capabilities of the two methods and compare the accuracy in the case of small track lengths as a function of sample size (*N*_s_). In this context, a single sample is defined as a pair of short trajectories: one contribution from a free filament and one from a bundle-embedded filament. For each sample size, we extract diffusion coefficients from 1500 individual repetitions, which we compile for distributions of diffusion coefficients and hence calculate means and SDs. We expect some likelihood that for small sample sizes, both methods will select a single-mode model. For large sample sizes, we expect that both methods will certainly select a two-mode model and accurately evaluate the diffusion coefficients associated with the distinct modes. In Fig. 5 *a*, we show the model selection success for the vbSPT method, expressed as the percentage of samples detected as bimodal. The detection success saturates for larger samples sizes. For small sample sizes, the model selection varies greatly depending on the strength of the diffusion coefficient prior *ρ*^*D*^, an input parameter to the Bayesian analysis scheme. In the small *N*_s_ limit, the dependence on *ρ*^*D*^ is nonmonotonic, which is evident when considering *N*_s_ = 1, as shown in Fig. 5 *b*. This indicates that for small samples, there is a limit to the model selection capability of the vbSPT method. To implement an a priori model selection scheme for using *H*(*η*), we fit both Eqs. 10 and 13 for each sample in a population and select for the minimal fitting residual with the data. We introduce a selection threshold *ϵ* to allow for a degree of filtering. When *R*_2_/*R*_1_ ≤ *ϵ*, where *R*_1_ and *R*_2_ are the fitting residuals for Eqs. 10 and 13, respectively, we impose a selection that is in favor of a single-mode model. In Fig. 5, *c* and *d*, we see that when *ϵ* = 1, we achieve a bimodal detection success of almost 100%. We can filter the data by varying *ϵ* and hence impose success rates equivalent to the vbSPT model. The peak success for the vbSPT model with the smallest sample size is ∼70% for *ρ*^*D*^ = 0.25. Setting *ϵ* = 0.95, we achieve an equivalent success rate. In Fig. 5 *e*, we compare the accuracy of the two diffusion coefficients extracted by each method for *ρ*^*D*^ = 0.25 and *ϵ* = 0.95 over a range of sample sizes for samples of two different trajectory lengths. For equivalent detection power, the accuracies of the two methods are in good agreement, with the vbSPT method exhibiting lower variation overall and a slightly more accurate evaluation of the slow mode. From this, we can confirm that our method is comparable with more sophisticated methods. In the case of larger data sets, the complexities associated with fitting and model selection can be avoided, and our method can be reduced to the essential features of peak identification in the logarithmic spectra. A key advantage here is the removal of the considerable implementation barriers required for sophisticated methods such as the vbSPT.

For one final example, in which we explore the generality of the method, we investigate the case of one-dimensional rotational diffusion, which is an intrinsic feature of our simulation system owing to our choice of anisotropic particles. We analyze the dynamics of the angle of filament orientation, which is given by the unit vector $\hat{u}$ in Eq. 15. This is an example of diffusion in one dimension with angular displacements *dθ*. For the uniform, monomodal systems, the probability density of the angular displacements *f*(*dθ*; *σ*) is the simplest form given by the generic density Eq. A1. We seek the probability density of *η*_*θ*_ = log_10_(*S*_*θ*_), with *S*_*θ*_ = *dθ*^2^/2Δ*t*. Following the general protocol, we map *dθ* → Θ such that *g*_Θ_(*dθ*) = *dθ*^2^ = Θ, with domain decomposition *dθ*_−_ ∈ [−*π*/2, 0], *dθ*_+_ ∈ (0, *π*/2). These finite domains, introduced because of periodicity, impose an upper limit on possible values of Δ*t*. Using the inverse transformations $g_{\text{Θ},\pm }^{-1}=\pm \sqrt{\text{Θ}}$ and derivatives $dg_{\text{Θ},\pm }^{-1}/d\text{Θ}=\pm 1/2\sqrt{\text{Θ}}$, we obtain the probability density

(17) $$F\left(\text{Θ};\sigma \right)=\frac{1}{\sigma \sqrt{2\pi \text{Θ}}}\text{exp}\left[-\frac{\text{Θ}}{2\sigma^{2}}\right].$$

The second transformation Θ → *η*_*θ*_ has inverse $g_{\eta_{\theta }}^{-1}=2\text{Δ}t10^{\eta_{\theta }}$ and derivative $dg_{\eta_{\theta }}^{-1}/d\eta_{\theta }=2\text{ln}\left(10\right)\text{Δ}t10^{\eta_{\theta }}$. It follows that the logarithmic measure of rotational diffusion is given by

(18) $$H\left(\eta_{\theta };\sigma \right)=\lambda_{\theta }\sqrt{10^{\eta_{\theta }}}\text{exp}\left[-\frac{\text{Δ}t10^{\eta_{\theta }}}{\sigma^{2}}\right],$$

where *λ*_*θ*_ = ln(10)$\sqrt{\text{Δ}t/\pi \sigma^{2}}$ and the rotational diffusion coefficient is *D*_*θ*_ = *σ*^2^/2Δ*t*. Likewise, it can be shown that the logarithmic measure for bimodal rotational diffusion is given by

(19) $$H\left(\eta_{\theta };\sigma_{1},\sigma_{2}\right)=\alpha \lambda_{\theta 1}\sqrt{10^{\eta_{\theta }}}\text{exp}\left[-\frac{\text{Δ}t}{\sigma_{1}^{2}}10^{\eta_{\theta }}\right]+\left(1-\alpha \right)\lambda_{\theta 2}\sqrt{10^{\eta_{\theta }}}\text{exp}\left[-\frac{\text{Δ}t}{\sigma_{2}^{2}}10^{\eta_{\theta }}\right],$$

where *λ*_*θ*1_ = ln(10)$\sqrt{\text{Δ}t/\pi \sigma_{1}^{2}}$ and *λ*_*θ*2_ = ln(10)$\sqrt{\text{Δ}t/\pi \sigma_{2}^{2}}$, with $\sigma_{1}^{2}$ = 2*D*_*θ*1_Δ*t* and $\sigma_{2}^{2}$ = 2*D*_*θ*2_Δ*t*.

**Figure 6:**
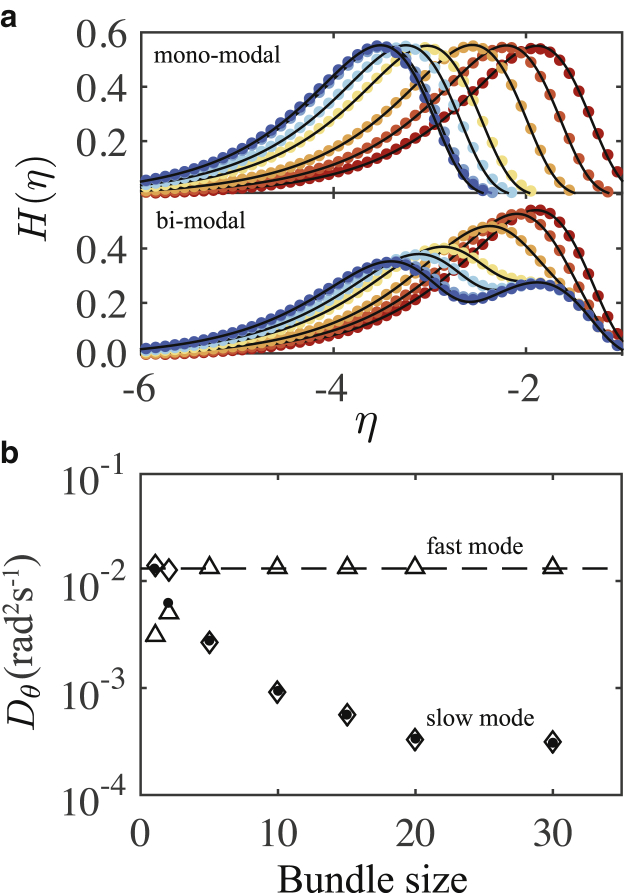
Logarithmic measure for 1D rotational diffusion of anisotropic filament bundles. (*a*) Monomodal (*top*) and bimodal (*bottom*) distributions on the *η* domain with fits of Eqs. 18 and 19 are given. The color scheme is the same as in Fig. 4*a*. (*b*) Extracted diffusion coefficients are shown. The dashed line is the exact value for a single filament. The symbol scheme is the same as in Fig. 4*b*. To see this figure in color, go online.

In Fig. 6 *a*, we show the distributions of *η*_*θ*_ from simulation data for monomodal and bimodal rotational diffusion, with the corresponding fits of Eqs. 18 and 19. The functional forms are accurate. Fig. 6 *b* shows the spectral decomposition of the diffusive modes, with the bold points representing the monomodal coefficients and the open symbols the bimodal coefficients. We have confirmed the presence of multiple diffusive and subdiffusive dynamic regimes on different timescales due to the cross-linking bond interactions. We consider the regime in which bundles rotate with collective rotational dynamics. It is difficult to compare with the corresponding MSD results because for timescales beyond ∼50 s, rotational displacements can be greater than *π* rads. This will introduce artifacts that affect the calculation of the linear MSD. However, for single filaments, we can compare experimental values to the theoretical value given by Eq. 14, which is *D*_*θ*_ = 0.0135 rad^2^ s^−1^. This value is indicated by the dashed line in the figure, indicating that the constant fast mode is accurate. Taken together, the results in Fig. 6 show that the method of extracting multimodal diffusion coefficients using the logarithmic measure is directly applicable to the rotational diffusion of anisotropic particles, further validating the generality of the method.

**Figure 7:**
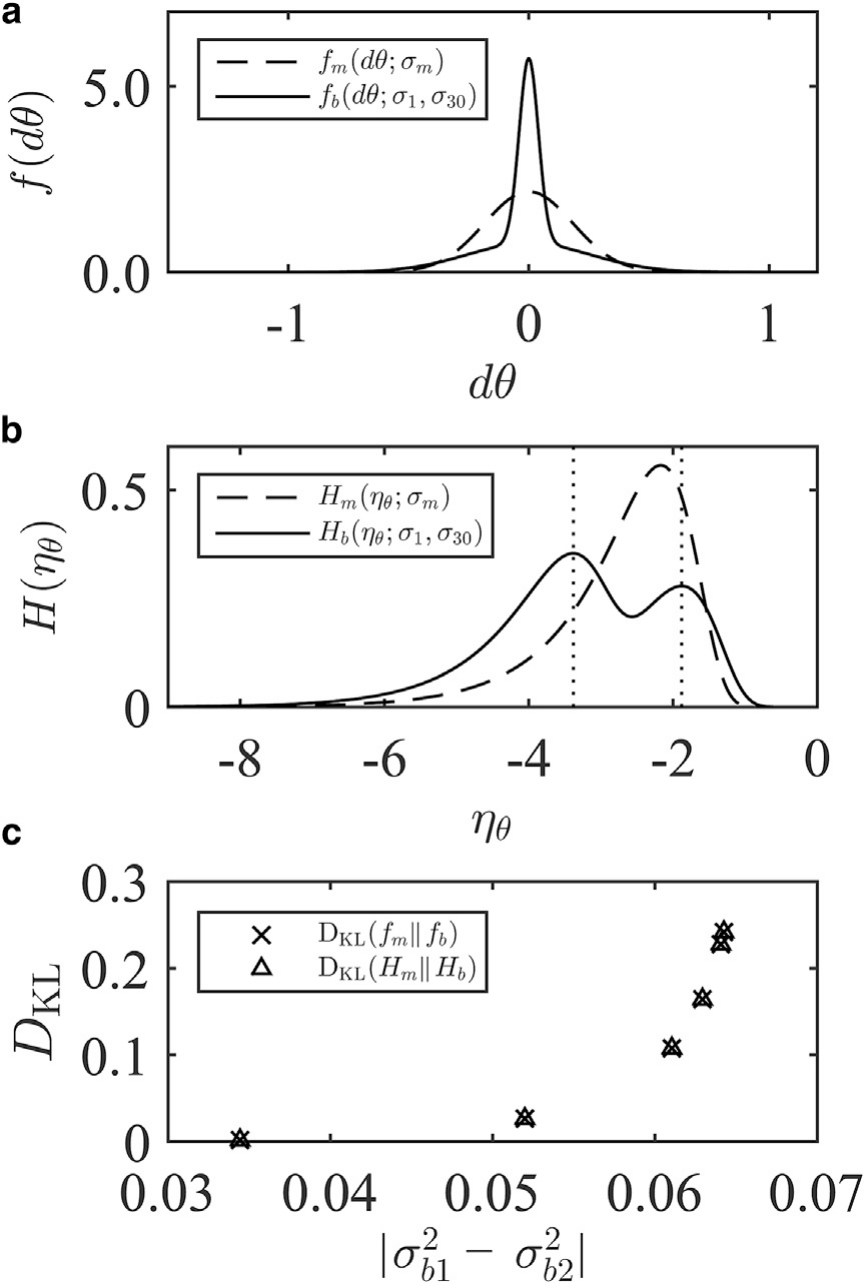
(*a*) Functional form of the probability density for a bimodal mixture of 1D rotational diffusion on the natural domain. *f*_*m*_ is a monomodal approximation for the known bimodal data with SD *σ*_*m*_. Parameters are *α* = 0.5, *σ*_*m*_ = 0.25 rad, *σ*_1_ = 0.18 rad, and *σ*_30_ = 0.04 rad. (*b*) Logarithmic measure of the same functions in (*a*) are shown. (*c*) KL divergence between bimodal and monomodal representations of known bimodal data is shown. The equivalence of the KL divergence confirms that the penalties incurred by misrepresenting the system modality are the same in the two domains, indicating no information loss for the logarithmic measure.

The 1D logarithmic measure for rotational diffusion also allows us to discuss the implications of the general method of representing Brownian data with the logarithmic measure. When the number of dimensions of the diffusion process is greater than 2, the scalar transform from displacements to *S* reduces the dimensionality of the analysis. The situation is otherwise in the case of 1D. Thus, in the case of 1D, we can consider the value of transforming to the logarithmic domain, rather than simply fitting a multimodal Gaussian sum in the natural domain. In Fig. 7, we show the probability densities of 1D, bimodal rotational diffusion. We take the variances from the values given in Fig. 6 *b* for bundle size 30, mixed with unbound filaments. We show the distribution of displacements on the natural scale *f*(*dθ*; *σ*_1_, *σ*_30_) and the same data transformed to the logarithmic domain *H*(*η*_*θ*_; *σ*_1_, *σ*_30_). In both representations, it is clear that there are multiple modes present. When analyzing in the natural domain, one would first guess the degree of modality and then fit the corresponding sum of Gaussians. For systems of lower signal/noise ratio, guessing the number of modes becomes difficult. Furthermore, fitting two parameters per mode becomes less reliable when the signal/noise ratio is low. When analyzing in the logarithmic domain, as in Fig. 7 *b*, the situation clarifies because one can read the number of modes from the number of peaks in the logarithmic spectrum. Moreover, as we have discussed, we can approximate the underlying diffusion coefficients from the locations of the peaks. Often, this approximation is sufficient, such that no further fitting is required. In the case of Fig. 7 *b*, the fast mode is located at *η*_*θ*_ = −1.88, indicating a diffusion coefficient of $D_{\theta_{1}}$ = 10^−1.88^ rad^2^ s^−1^ = 0.0135 rad^2^ s^−1^. The slow mode is located at *η*_*θ*_ = −3.38, indicating $D_{\theta_{2}}$ = 10^−3.38^ rad^2^ s^−1^ = 4.16 × 10^−4^ rad^2^ s^−1^. As expected, these results agree well with Fig. 6.

**Figure 8:**
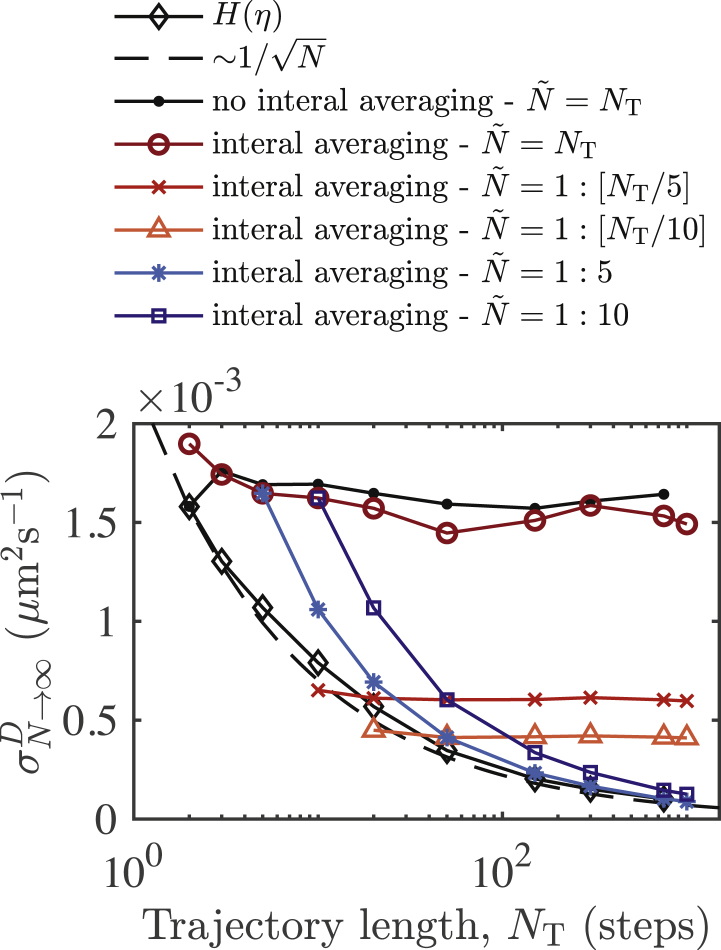
Errors in the population ensemble diffusion coefficient depend on MSD-length selection. In contrast to the 1/$\sqrt{N_{\text{T}}}$ scaling for the length dependence of the MSD and diffusion coefficient error in single-particle trajectories, the population errors can be constant over a range of trajectory lengths. Depending on the range of time lags of the MSD used for the calculation of the diffusion coefficient, the errors can approach the 1/$\sqrt{N_{\text{T}}}$ scaling exhibited by the *H*(*η*) method. To see this figure in color, go online.

In addition to ensuring that the transformations provide a practical readout of the spectra of diffusion coefficients, it is also important to ensure that there is no loss of information or accuracy when switching to the logarithmic domain. We do this by comparing the Kullback-Leibler (KL) divergences between the known bimodal representations of bimodal data, *f*(*dθ*; *σ*_1_, *σ*_30_) and *H*(*η*_*θ*_; *σ*_1_, *σ*_30_), and monomodal approximations, *f*(*dθ*; *σ*_*m*_) and *H*(*η*_*θ*_; *σ*_*m*_), that are known to incorrectly under-represent the modality of the data. The KL divergence tells us how well one density function can represent another. By calculating the KL divergence between the bimodal representations and the monomodal representations of data that are known to contain a bimodal signal, we can quantitatively compare the penalty of misrepresenting the underlying modality in the two domains. If the penalty is less in the logarithmic domain, then information has been lost in the transformation, implying that it is quantitatively more difficult to represent the correct modality of the system in this domain. We also show the monomodal approximations of the bimodal data in Fig. 7, *a* and *b*. *σ*_*m*_ is the SD calculated in the *dθ* data for the bimodal systems given in Fig. 6. We provide the details for the calculation of the KL divergences in Appendix C. We plot the values of the KL divergence for each of the bimodal pairs in Fig. 7 *c*. For all bimodal mixtures, the KL divergences in both domains are equivalent. Thus, we can quantitatively confirm that there is no loss of information incurred when representing the logarithmic measure of a Brownian process.

## Conclusions

The diversity of both living and inanimate systems investigated using SPT/SMT ensures a need for the ongoing contribution of novel analysis tools. On the one hand, tools that employ complex analysis pipelines will take us deeper into understanding novel molecular processes. On the other hand, simple and easy-to-implement tools allow fast access to characterizing novel systems, with benefits to engineers and applied scientists. The method presented in this work is an example of the latter. It is easy to implement and is suitable for the evaluation of the nonuniform dynamics present in mixed, diffusive systems. Recent years have seen the application of the method in the context of SPT with initial successes. Here, we have provided a theoretic foundation for the method. With this theoretical foundation at hand, we could quantitatively benchmark the analysis pipeline of transforming mixed Brownian data to the logarithmic domain and subsequently calculating the probability density. We provide analytical functions that can be fitted to the data for an accurate evaluation of the underlying diffusion coefficients. Alternatively, one can simply display the spectra and visually identify the modes by indicating the distinct spectral peaks. Either approach reveals whether the Brownian dynamics of the underlying molecular process is multimodal, without the need for prior knowledge of the molecular states or species identities. We can extend the analysis to systems of any dimensionality and to systems with any number of diffusive modes, which we have verified by considering the anisotropy inherent in our choice of simulation model.

There are limitations to the method. For example, the difference in the diffusion coefficients for different modes must be greater than a critical difference to detect the distinct modes. There also exists some critical ratio of population concentrations beyond which one population cannot be detected. Furthermore, we have made no mention of the treatment of measurement errors. The purpose of this work is to provide a rigorous theoretical foundation to establish the logarithmic measure of Brownian data, without taking into account measurement errors such as localization error or motion blur. The impact of the latter changes with technological developments, whereas the former is an essential component of the phenomena themselves. Our importance is placed on clarifying the fundamentals in the phenomena that are independent of measurement technologies.

Finally, it is important to note that many biological systems exhibit anomalous diffusion. This is especially so in the context of filamentous gels, which form the simulation basis of this article. Here, we have restricted our attention to Brownian systems with Gaussian distributed displacements for the sake of providing a complete and thorough analysis. The applicability of our approach to systems outside the scope of normal diffusion is also of interest from both application and theoretical points of view. In fact, the usefulness of our approach itself has been demonstrated empirically through the analyses of complex phenomena dating back to its proposal (4) in which the molecules of interest exhibit adsorption, and also in a recent application (5) in which we analyze strongly confined yet excited systems. It is, however, nontrivial to address this applicability within the theoretical context of this work. The theoretical basis of this work is placed on the Gaussianity of the displacement distribution. At least, it can be said that subdiffusive systems can exhibit Gaussian displacement distributions as well. As far as the timescale of confinement is limited, the use of a sufficiently long time span yields Gaussian displacement distributions, as ensured by the central limit theorem. Nevertheless, there can be diverse specifications in the category of subdiffusive systems. Such an approach may involve the moment scaling spectrum or the calculation of Hurst exponents (46). It is therefore nontrivial to make a brief conclusion universally on the relations between an ensemble formalism and the time-dependent characteristics of a system—for example, to either subdiffusive or superdiffusive systems—and that is therefore beyond the scope of this work.

## Author contributions

Conceptualization, I.H.; methodology, B.A.D., I.F.S., and I.H.; simulation software, B.A.D. and I.F.S.; formal analysis and investigation, B.A.D. and I.H.; resources, I.F.S. and I.H.; data curation, B.A.D. and I.F.S.; writing—original draft, B.A.D.; writing—review and editing, I.F.S., and I.H.; visualization, B.A.D.; supervision and project administration, I.F.S. and I.H.; funding acquisition, B.A.D., I.F.S., and I.H.

## Appendix A: generalized protocol for the derivation of the logarithmic measure of normal processes

We outline a protocol for deriving analytic forms of the logarithmic measure of any *n*_d_-dimensional, multimodal, normal (i.e., not anomalous) process:

1) Identify the correct form for the probability density of the displacements in each dimension. For normal processes, the general form is

(A1) $$f\left(dx_{i};\sigma_{1},\ldots ,\sigma_{M}\right)=\overset{M}{\underset{k=1}{\sum }}\frac{a_{k}}{\sigma_{k}\sqrt{2\pi }}\text{exp}\left[-\frac{dx_{i}^{2}}{2\sigma_{k}^{2}}\right],$$

where $\sigma_{k}^{2}$ is the variance of the *k*^th^ mode in the *i*^th^ dimension. Here, *a*_*k*_ is the modal weighting factor where $\sum_{k=1}^{M}a_{k}$ = 1. We assume that the set of *M* variances is the same in each dimension.
2) Establish a new variable *Z*, which is the multivariate transformation of the displacements in *n*_d_ dimensions to a single scalar value:

(A2) $$Z\left(dx_{1},\ldots ,dx_{n_{\text{d}}}\right)=\overset{n_{\text{d}}}{\underset{i=1}{\sum }}dx_{i}^{2}.$$

Subsequently, we introduce the diffusion element *S*:

(A3) $$S\left(dx_{1},\ldots ,dx_{n_{\text{d}}};\text{Δ}t\right)=\frac{Z}{2n_{\text{d}}\text{Δ}t}.$$

The aim of the derivation outlined by this protocol is to find the appropriate form for the probability density of log_10_(*S*), which we refer to, in general, as the logarithmic measure.
3) Transform variables *dx*_*i*_ → *X*_*i*_ such that $g_{X_{i}}\left(dx_{i}\right)=dx_{i}^{2}$ = *X*_*i*_, where $g_{X_{i}}$ is the transformation with defined inverse. Determine the inverse transformations $g_{X_{i}}^{-1}$ and their derivatives $dg_{X_{i}}^{-1}/dX_{i}$. In general, each transformation is a many-to-one mapping and cannot be uniquely inverted. Make them invertible by defining one-to-one transformations in the positive and negative domains *dx*_*i*,−_ ∈ (−∞, 0], *dx*_*i*,+_ ∈ (0, ∞) with inverse transformations $g_{X_{i},\pm }^{-1}=\pm \sqrt{X_{i}}$ and derivatives $dg_{X_{i},\pm }^{-1}/dX_{i}=\pm 1/2\sqrt{X_{i}}$.
4) Transform the probability densities over the decomposition of domains *f*(*dx*_*i*_; *σ*_1_, …, *σ*_*m*_) → *F*(*X*_*i*_; *σ*_1_, …, *σ*_*m*_):

(A4) $$F\left(X_{i};\sigma_{1},\ldots ,\sigma_{m}\right)=\underset{j=+,-}{\sum }f\left(g_{X_{i},j}^{-1};\sigma_{1},\ldots ,\sigma_{m}\right)\left|\frac{dg_{X_{i},j}^{-1}}{dX_{i}}\right|.$$

and hence determine *F*(*Z*; *σ*_1_, …, *σ*_*m*_).
5) Establish a new variable *η* = log_10_(*S*). Then *η* is the new independent variable.
6) Transform the random variable *Z* → *η* such that *g*_*η*_(*Z*) = *η* = log_10_(*Z*) − log_10_(2*n*_d_Δ*t*). Determine the inverse of the transformation $g_{\eta }^{-1}$ = 2*n*_d_Δ*t*10^*η*^ and its derivative $dg_{\eta }^{-1}$/*dη* = 2*n*_d_Δ*t*ln(10)10^*η*^.
7) Transform the probability densities *F*(*Z*) → *H*(*η*) to find the probability density of *η*:

(A5) $$H\left(\eta \right)=F\left(g_{\eta }^{-1}\right)\left|\frac{dg_{\eta }^{-1}}{d\eta }\right|.$$

*H*(*η*) is the logarithmic measure of diffusion.

## Appendix B: a comparison of population ensemble approaches to evaluating errors in the single-mode diffusion coefficient from MSDs

We consider various ensemble approaches to evaluating the diffusion coefficient via the MSD. In particular, we consider the errors associated with each approach. The choice is somewhat arbitrary. However, the resultant errors are dramatically affected. Because we benchmark the errors associated with the *H*(*η*) method against the MSD approach, it is important to clearly indicate these possibilities.

We note the distinction between the population ensemble approach and the length-dependent single-particle approach. The latter is discussed in detail by Qian et al. (45). It is an estimate of the error in the diffusion coefficient from the MSD for a single trajectory, which scales as ∼1/$\sqrt{N_{\text{T}}}$ because of internal averaging over the full set of trajectory time lags (3). For the ensemble approaches, internal averaging may or may not be applied. For a single trajectory of length *N*_T_, we choose an arbitrary sublength $\tilde{N}$ ≤ *N*_T_ and apply linear regression to the first $\tilde{N}$ elements of the MSD. The gradient of the linear fit provides the diffusion coefficient. We evaluate the diffusion coefficient in this way over a population of *N*_s_ individual trajectories resulting in a distribution of diffusion coefficients.

In Fig. 8, we show the SD as a function of trajectory length *N*_T_ for various population ensemble calculations with *N*_s_ = 3000 samples. The figure legend indicates when internal averaging is applied. The various curves represent different choices for $\tilde{N}$. When we use the full MSD ($\tilde{N}$ = *N*_T_) to calculate the linear regression then the inclusion of internal averaging does not influence the errors, which are constant in either case. This result is in stark contrast to the expected ∼1/$\sqrt{N_{\text{T}}}$ scaling of Qian et al. (45) for a single trajectory, which is a direct result of internal averaging. Note that when we set $\tilde{N}$ to a small value, irrespective of *N*_T_, then the population ensemble *σ*^*D*^ approaches ∼1/$\sqrt{N_{\text{T}}}$ scaling.

## Appendix C: details of the KL divergence

In 1D, a monomodal Gaussian with variance $\sigma_{m}^{2}$ and zero mean is given by

(C1) $$f_{m}\left(x;\sigma_{m}\right)=\frac{1}{\sigma_{m}\sqrt{2\pi }}\text{exp}\left[-\frac{x^{2}}{2\sigma_{m}^{2}}\right].$$

The variance $\sigma_{m}^{2}$ is calculated using the standard variance formula, applied to the simulation data for known bimodal distributions. Then, $\sigma_{m}^{2}$ represents a reduced-modal approximation to the data, which we know to be inaccurate.

A bimodal Gaussian mixture characterized by two variances $\sigma_{\text{b}1}^{2}$ and $\sigma_{\text{b}2}^{2}$ is given by

(C2) $$f_{b}\left(x;\sigma_{b1},\sigma_{b2}\right)=\frac{\alpha }{\sigma_{b1}\sqrt{2\pi }}\text{exp}\left[-\frac{x^{2}}{2\sigma_{b1}^{2}}\right]+\frac{\left(1-\alpha \right)}{\sigma_{b2}\sqrt{2\pi }}\text{exp}\left[-\frac{x^{2}}{2\sigma_{b2}^{2}}\right],$$

where $\sigma_{\text{b}1}^{2}$ and $\sigma_{\text{b}2}^{2}$ are calculated from simulations, as described in the main text.

Transformation of Eq. C1 to the logarithmic domain gives

(C3) $$H_{m}\left(\eta ;\sigma_{m}\right)=\frac{\text{ln}\left(10\right)}{\sigma_{m}}\sqrt{\frac{10^{\eta }}{\pi \text{Δ}t}}\text{exp}\left[-\frac{\text{Δ}t10^{\eta }}{\sigma_{m}^{2}}\right],$$

where the independent variable is *η* = log_10_(*x*^2^/Δ*t*). Likewise, the logarithmic measure for the bimodal mixture is

(C4) $$\begin{array}{c} H_{b}\left(\eta ;\sigma_{b1},\sigma_{b2}\right)=\frac{\text{ln}\left(10\right)}{2\sigma_{b1}}\sqrt{\frac{10^{\eta }}{\pi \text{Δ}t}}\text{exp}\left[-\text{Δ}t\frac{10^{\eta }}{\sigma_{b1}^{2}}\right]\\ \;+\frac{\text{ln}\left(10\right)}{2\sigma_{b2}}\sqrt{\frac{10^{\eta }}{\pi \text{Δ}t}}\text{exp}\left[-\text{Δ}t\frac{10^{\eta }}{\sigma_{b2}^{2}}\right], \end{array}$$

where we have used the mixing parameter *α* = 1/2. The variances $\sigma_{m}^{2}$, $\sigma_{b1}^{2}$, and $\sigma_{b2}^{2}$ are the same is in Eqs. C1 and C2.

To numerically approximate the KL divergence for a pair of probability densities, we use the following: for a normal process on the natural scale,

(C5) $$D_{\text{KL}}\left(f_{m}\left|\right|f_{b}\right)=\underset{x\in {\text{Λ}}_{x}}{\sum }f_{m}\left(x;\sigma_{m}\right)\text{log}\left(\frac{f_{m}\left(x;\sigma_{m}\right)}{f_{b}\left(x;\sigma_{b1},\sigma_{b2}\right)}\right)dx,$$

where Λ_*x*_ is the domain over which we have sampled the displacements. For the logarithmic measure,

(C6) $$D_{\text{KL}}\left(H_{m}\left|\right|H_{b}\right)=\underset{\eta \in {\text{Λ}}_{\eta }}{\sum }H_{m}\left(\eta ;\sigma_{m}\right)\text{log}\left(\frac{H_{m}\left(\eta ;\sigma_{m}\right)}{H_{b}\left(\eta ;\sigma_{b1},\sigma_{b2}\right)}\right)d\eta ,$$

where Λ_*η*_ is the transformed domain of displacements.
